# Characterization of the Phenolic Compounds in Different Plant Parts of *Amaranthus* *cruentus* Grown under Cultivated Conditions

**DOI:** 10.3390/molecules25184273

**Published:** 2020-09-18

**Authors:** Tlou Grace Manyelo, Nthabiseng Amenda Sebola, Zahra Mohammed Hassan, Monnye Mabelebele

**Affiliations:** 1 Department of Agriculture and Animal Health, College of Agriculture and Environmental Sciences, University of South Africa, Florida 1710, South Africa; manyelo.t.g@gmail.com (T.G.M.); sebolan@unisa.ac.za (N.A.S.); zahrabattal@gmail.com (Z.M.H.); 2 Department of Agricultural Economics and Animal Production, University of Limpopo, Sovenga 0727, South Africa

**Keywords:** amaranth, phenolics, growth stage, metabolites, antioxidants

## Abstract

Phenolic compounds that are present in amaranth crops have gained a lot of interest from researchers due to their health benefits potential. Therefore, the aim of this study was to investigate phenolic compounds present in different plant parts of *Amaranthus* *cruentus* using liquid chromatography-electrospray ionization quadrupole time-of-flight mass spectrometry. Moreover, data were analyzed by one-way analysis of variance of the statistical analysis software, whereas commercial statistical package version 4.02 was used for principal component analysis. A total of 21 phenolic compounds were detected and eight were not identified. Caffeoylsaccharic acid isomer, coumaoryl saccharic acid, tryptophan, feruloyl-d-saccharic acid isomer a, b, and c, caffeoyl isocitrate, quercetin 3-*O*-rhamnosyl-rhamnosyl-glucoside, feruloyl isocitrate, hyperoside, kaempferol rutinoside, and alkaloid compounds were mostly detected in tender and mature leaves. Generally, rutin content was higher (*p* < 0.05) in most vegetative parts of the amaranth plant, thus, late maturity leaves, tender leaves, and mature leaves, respectively. Lower quantities of rutin were observed in tender grains, flowers, and mature grains. It can be concluded that amaranth contains phenolic compounds, predominantly in the vegetative parts, which makes it to be a promising source of phenolic compounds beneficial to human health.

## 1. Introduction

The quest to search for bioactive compounds of natural origin will always remain a core objective of many researchers. It is believed that finding these compounds will not only improve household nutrition but will also improve the standard of living and general public health [1,2]. To date, several research papers have indicated that plant-derived polyphenols have anti-aging, anti-inflammatory, and anti-proliferative properties [3,4] and, moreover, have been shown to help reduce the risk of chronic illnesses [2]. A thriving polyphenol market is estimated to increase due to recent demand and market size [5]. Further to this, it is indicated that global demand for polyphenols in 2025 is expected to reach USD 1904.1 million [5], which is a clear indication of how important these compounds are. It is now known that the health-promoting effects of polyphenols are broader than their basic antioxidant function. Polyphenols have been shown to control aging and degenerative diseases due to their ability to inhibit cyclooxygenases and lipoxygenaseenzyme enzymes involved in inflammation [6] or acetylcholinesterase [7], associated with neurodegenerative diseases.

*Amaranthus* is one of the crops that has gained the most attention because of its endowment with phenolic compounds [8,9,10,11]. Amaranth forms part of the diets and incomes of households in most parts of the world such as America, Africa, India, and Nepal.

*Amaranthus cruentus* is known as an annual herbaceous plant with a short growing period ranging between 4–6 weeks. It reproduces only by seeds and results in one large central root known as a tap root, followed by thick ribbed and red-dyed stems of 0.1 to 2.0 m in height, which are often straight and branched [12]. Leaf shapes vary from ovate to rhombic-ovate arranged spirally, simple without stipules. The plant produces numerous green flowers and forms long finger-like spikes with a dense terminal panicle and axillary spikes below [13]. Flowers have five sharp and long tip tepal segments that are 2–3 mm long and which cause the plant inflorescence to feel prickly. At maturity, the plant may be characterized by a high reddish color variability with inflorescences, which are about 50 cm in height [14]. Each of these inflorescences produces more than 50,000 seeds with white, pink, and brown to black color variability and that normally appear round in shape, while some are often lenticular in shape with a smooth and thin coat. Its seeds size range between 1 to 1.5 mm in diameter, and with 1000 seed mass of 0.6–1.0 g [12]. Its leaves and seeds are known to provide adequate amounts of nutrients such as protein, vitamins, minerals, dietary fiber [15]. The proteins are known to be rich in essential amino acids that cannot be found in other cereals and that is why they are regarded as of high nutritional quality [1]. It is indicated that different plant parts (flowers and stalks) and leaves contain phytochemicals [3,16] which serve as potential nutrient in human and animal diets [10]. Moreover, the consumption of *Amaranth cruentus* products generally plays a significant role in patients suffering from celiac disease and diabetes. Several studies have been conducted on the value of amaranth, including secondary metabolites with beneficial effects and pharmacological properties [3,4]. Recently, antioxidants present in plants such as the amaranth crop have gained a lot of interest from researchers due to their potential health beneficial effects, such as fighting against chronic diseases like cardiovascular diseases, cancer, cataracts, just to mention a few [1,14,17,18]. Amaranth has been reported to contain several phenolic compounds such as hydroxycinnamic acids, benzoic acids, flavonoids, and alkaloids, which has been shown to be influenced by plant stage of maturity, soil conditions, fertilizers, and moisture availability [8,9]. In South Africa, the leafy amaranth, together with other green leaves, are locally known as morogo (Tswana for vegetables) [14]. The leaf is the most consumed part of the plant and usually occurs as a volunteer crop during the rainy season [19]. Some information exists on the phenolic compounds of amaranth; however, very scarce information exists on the amaranth grown in South Africa. It is therefore, imperative to study phenolic compounds of different amaranth plant parts to clearly document the present of phenolic compounds in different growth stages of amaranth. Hence, the aim of this study was to investigate phenolic compounds present in other plant parts at different growth stages of *Amaranthus cruentus* grown under South African climatic conditions using liquid chromatography-electrospray ionization quadrupole time-of-flight mass spectrometry.

## 2. Results and Discussion

### 2.1. Phenolic Compounds

**Figure 1:**
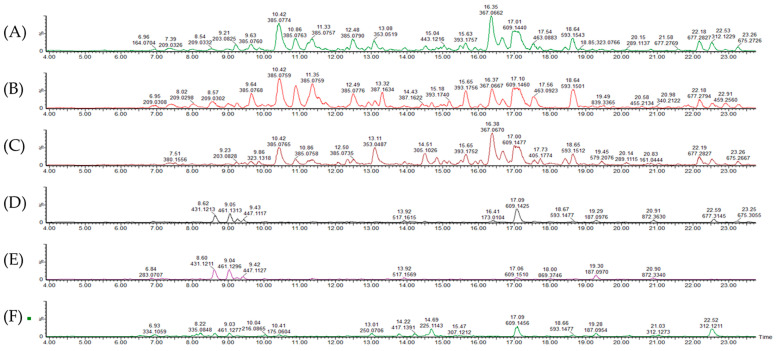
Chromatograms of (**A**) tender leaves, (**B**) mature leaves, (**C**) late maturity leaves, (**D**) mature grains, (**E**) tender grains, and (**F**) flowers.

**Figure 2:**
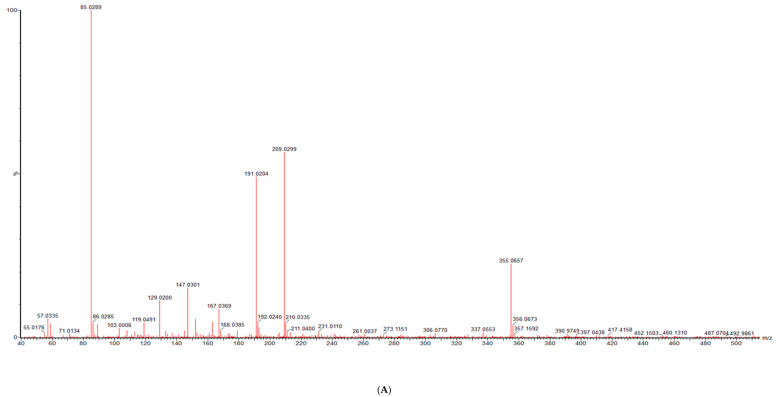

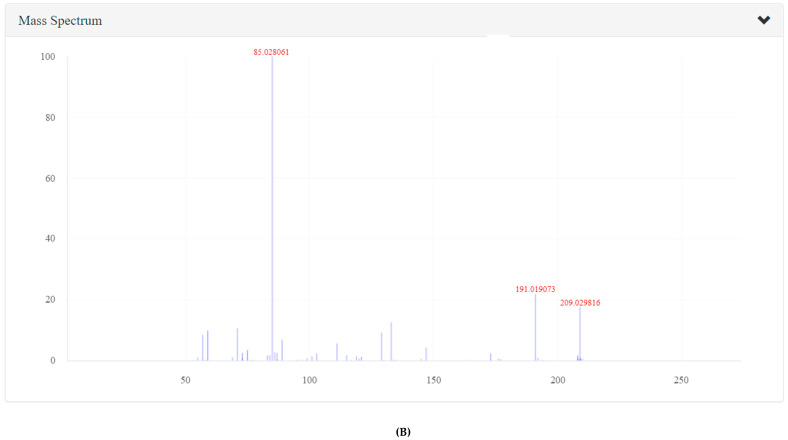
The LC-MS characterization of coumaoryl saccharic acid; (**A**) a chromatograph of p-coumaoryl saccharic acid (compound **5**, Table 1), retention time (RT = 8.98 min) in the negative mode of ionization (M − H)^−^ tentatively identified in tender leaves, mature leaves, late maturity leaves, mature grains and flowers and not found in tender grains; (**B**) MS/MS spectrum of coumaoryl saccharic acid reflecting the product ion of *m*/*z* 93, confirmation via online LC-MS library and database.

The characterization of the phenolic profiles of different plants has been widely determined using LC-MS [20]. The phenolic compounds identified in amaranth plant parts using LC-MS methods are presented in Table 1, and chromatograms of different growth stages of the amaranth plant are presented in Figure 1. In the present study, about 21 compounds were detected with eight unknown compounds shown in Table 1. Compound **1** was identified as caffeoylsaccharic acid isomer based on precursor ion [M − H]^−^ at *m*/*z* 371.063 with product ions at *m*/*z* 415, 371, 283, 209, and 151 with 0.8 mass error (ppm). It is worth mentioning that this acid was not quantified in the grains and flowers, as shown in Table 2. Caffeoyl derivatives were previously reported as being abundant in tomato cotyledons by Strack et al. [21] and were also recently recorded in amaranth by Karamać et al. [8] and Neugart et al. [16]. Compound **5,** having a precursor ion [M − H]^−^ at *m*/*z* 355.069 with product ions at *m*/*z* 209.191 and 147.85 was tentatively identified as coumaoryl saccharic acid and was present in tender leaves, mature leaves, late maturity leaves, mature grains and flowers and not found in tender grains. This compound **5** showed a strong saccharic acid spectrum with ions at 209.0297, 191.0196, 147.0274, 133.0167, 85.0288 typical of saccharic acid (Figure 2). This leaves a gap of C9H8O3, which corresponds to coumaric acid. So, the compound was proposed to be an ester of coumaric and saccharic acids. A previous study reported that raw *A. caudatus* grains had derivatives of coumaric acids compounds [22]. Tryptophan and feruloyl-d-saccharic acid isomer a (Compound **6**, [M − H]^−^ at *m*/*z* 203.82, and compound **8**, [M − H]^−^ *m*/*z* 385.077) were present in amaranth tender leaves, mature leaves, late maturity leaves, mature grains, tender grains, and flowers. However, product ions for tryptophan compound were not detected. Whereas molecular ions for feruloyl-d-saccharic acid isomer a produced products ions at *m*/*z* 209, 191, and 147.85. Feruloyl-d-saccharic acid isomer b (Compound **9**, [M − H]^−^ *m*/*z* 385.078) and feruloyl-d-saccharic acid isomer c (Compound **10**, [M − H]^−^ *m*/*z* 385.077) were present in amaranth tender leaves, mature leaves, late maturity leaves, and flowers and not found in tender and mature grains. Feruloyl-d-saccharic acid isomer compounds were noted to have been recorded in three different retention times, at subsequent [M − H]^−^ this could be explained that the system has repeatedly picked the same compound more than once. Compound **11** was identified as caffeoyl isocitrate based on precursor ion [M − H]^−^ at *m*/*z* 353.053 with product ions at *m*/*z* 191, 173, 155, and 111. This compound was present in tender leaves, mature leaves, late maturity leaves, and mature grains but not present in tender grains and flowers. Caffeoyl isocitrate was previously reported in leaves, seeds, and other aerial parts of *A. caudatus* [16,23]. Quercetin 3-*O*-rhamnosyl-rhamnosyl-glucoside (Compound **15** with [M – H]^−^ at *m*/*z* 755.205), yielding product ions at *m*/*z* 611, 300, 271 was identified and found to be present in tender leaves, mature leaves, late maturity leaves, mature grains and not found in tender grains and flowers. Compound **16** was tentatively identified as feruloyl isocitrate with parent ion [M – H]^−^ at *m*/*z* 367.068 with product ions at *m*/*z* 173, 154, and 111. This compound was present in tender leaves, mature leaves, late maturity leaves, mature grains, tender grains, and flowers. Its highest peak was recorded in tender leaves. This feruloyl compound derivatives were previously reported in *A. caudatus* in leaves, seeds, and other aerial parts of amaranth [16,24]. The identification of rutin (Compound **17**) was confirmed based on precursor ion [M − H]^−^ at *m*/*z* 609.143 with product ions at *m*/*z* 300 and 271. Rutin compound was found to be present in tender leaves, mature leaves, late maturity leaves, mature grains, and tender grains and flowers. In a previous study, Kalinova and Dadakova [23] found rutin contents in seeds and flowers of six *Amaranthus* spp. including *A. cruentus* species; the authors stated that rutin content in seeds and flowers varied according to the developmental stages of the crop and that is usually increased with plant aging. Compound **18** was identified as quercetin galactoside based on precursor ion [M – H]^−^ at *m*/*z* 463.086 with product ions at *m*/*z* 300 and 271. Quercetin galactoside was found to be present in tender leaves, mature leaves, late maturity leaves, mature grains, and flowers and not found in tender grains. According to Kalinova and Dadakova [23], amaranth cruentus species contain quercetin or its derivatives. Compound **19** was tentatively named as Kaempferol-3-*O*-rutinoside based on precursor ion [M − H]^−^ at *m*/*z* 593.151 with product ions at *m*/*z* 285 and 255. Kaempferol-3-*O*-rutinoside compound was found to be present in tender leaves, mature leaves, late maturity leaves, mature grains, and tender grains and flowers. Kaempferol-3-*O*-rutinoside contents were previously detected in amaranth leaves, seeds, and other aerial parts [16,21]. Compound **21** was detected in tender leaves, mature leaves, late maturity leaves, mature grains, and tender grains and flowers and was tentatively identified as alkaloid according to the precursor ions [M − H]^−^ at *m*/*z* 677.283 and 312.124, with products ions at 645, 617, 489, 279, 152, and 190, 178, 148 and 135, respectively. Previously, it was reported that amaranth leaves are rich in alkaloids [25] with various health-promoting properties such as free radical scavengers that prevent oxidative cell damage and have strong anticancer activities [26]. Whereas Olowolaju et al. [27] reported that alkaloids were present at all amaranth stages of growth.

### 2.2. Quantified Phenolic Compounds in Amaranth

**Figure 3:**
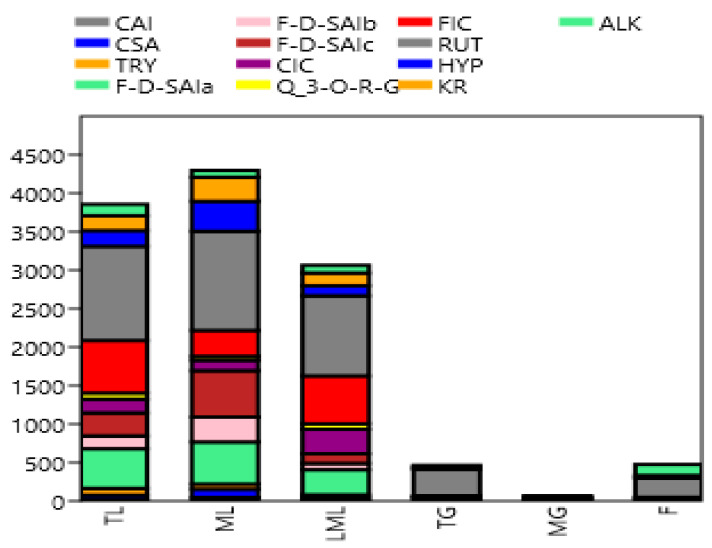
Stacked bar graph of phenolic compounds in amaranth plant (mg/kg). CAI, caffeoylsaccharic acid isomer; CSA, coumaoryl saccharic acid; TRY, tryptophan; F-d-SAIa, feruloyl-d-saccharic acid isomer a; F-d-SAIb, feruloyl-d-saccharic acid isomer b; F-d-SAI c, feruloyl-d-saccharic acid isomer c; CIC, caffeoyl isocitrate; Q 3-*O*-RG, quercetin 3-*O*-rhamnosyl-rhamnosyl-glucoside; FIC, feruloyl isocitrate; RUT, rutin; HYP, hyperoside; KR, kaempferol rutinoside; ALK, alkaloid; TL, Tender leaves; ML, mature leaves; LML, late maturity leaves; TG, tender grains; MG, mature grains; F, flowers.

Quantified individual phenolic compounds are shown in Table 2 and Figure 3. Amaranth mature leaves showed higher (*p* < 0.05) caffeoylsaccharic acid isomer and coumaoryl saccharic acid contents than tender leaves, late maturity leaves, tender grains, mature grains, and flowers. However, previous studies reported high caffeoyl and coumaoryl dervatives in amaranth grains [22,28]. Caffeolyl and coumaoryl have previously been reported to be useful in treating kidney pains, gastrointestinal inflammation, and to kill intestinal parasites [29]. Tender amaranth leaves showed higher (*p* < 0.05) tryptophan contents than mature leaves, late maturity leaves, tender grains, mature grains, and flowers. However, Soriano-García and Aguirre-Díaz [30] reported amaranth grains to be rich in tryptophan. This compound is known as an essential component of the diet, which plays an important role in protein synthesis, and it is a precursor of biologically active compounds such as serotonin, melatonin, quinolinic acid. Several studies have shown that tryptophan supplementation in the daily diet intake of human beings might improve pharmacotherapy in some carcinogenesis diseases [31]. Mature leaves exhibited higher (*p* < 0.05) feruloyl-d-saccharic acid isomer a, b, and c than tender leaves, late maturity leaves, tender grains, mature grains, and flowers, while late maturity leaves showed higher (*p* < 0.05) caffeoyl isocitrate contents than amaranth tender leaves, mature leaves, tender grains, mature grains, and flowers. Amaranth tender leaves showed higher (*p* < 0.05) quercetin 3-*O*-rhamnosyl-rhamnosyl-glucoside and feruloyl isocitrate than mature amaranth leaves, late maturity leaves, tender grains, mature grains, and flowers. However, Kalinova and Dadakova [23] found that amaranth leaves at the maturity stage contained high quercetin or its derivatives than during their early stage of maturity. Rutin content was higher (*p* < 0.05) in the tender, mature late maturity leaves as it ranges from 1041.9, 1222.8, and 1289.8 mg/kg, respectively. Lower (*p* > 0.05) quantities of rutin were registered in tender grains, flowers, and mature grains, which ranged from 26.4, 342.2, and 252.2 mg/kg, respectively. Although grain amaranths are considered high in manifold nutraceutical properties, as stated in Caselato-Sousa and Amaya-Farfan [32], the leaves in this study are endowed with higher quantities of the phenolics. This variation might be attributed to the complex interaction between phenolic acids and the cell wall elements of the grains. It was also observed during this study that rutin seems to be the most common phenolic compound found in amaranth, as many studies have documented its presence [8,23,28]. In addition, it was also found to be the most abundant phenolic compounds in amaranth in the current study. Karama’c et al. [8], Khanam and Oba [33], and Kalinova and Dadakova [23] also found rutin to be the most plentiful phenolic compound in amaranth leaves. However, Vollmannová et al. [34] found the quantity of rutin in amaranth (*A. hypochondriacus*) seeds to be higher with the range of 310–508 mg/kg DM, though the quantity in the current study was much higher. The reason for this variation might be due to different amaranth species (*A. cruentus*) used in the present study. Rutin is one of the phytochemical compounds which are important in protecting plants against different environmental stress conditions such as wounding, infection, and excessive light or UV irradiation [35,36,37]. Kwon et al. [38] also reported on the importance of rutin in the prevention and treatment of colorectal carcinogenesis in human beings. Mature leaves showed higher (*p* < 0.05) quercetin galactoside and kaempferol rutinoside contents than tender leaves, late maturity leaves, tender grains, mature grains, and flowers. Kaempferol rutinoside has been reported to decrease the severity of arthritic inflammation, protecting joints from degradation [39]. Tender leaves exhibited higher (*p* < 0.05) alkaloid contents than mature, late maturity leaves, tender grains, mature grains, and flowers. The results of the present study are consistent with the results of [25], who reported that amaranth leaves are rich in alkaloids. Moreover, Olowolaju et al. [27] reported that alkaloids were present at all amaranth stages of growth. Alkaloids are known to have health-promoting properties such as free radical scavengers that prevent oxidative cell damage and have strong anticancer activities [26]. The chromatograph figures clearly demonstrated the high presence of the phenolic compounds in the leafy parts in comparison to the other parts, as shown by the high and low peaks.

### 2.3. Principal Component Analysis (PCA)

**Figure 4:**
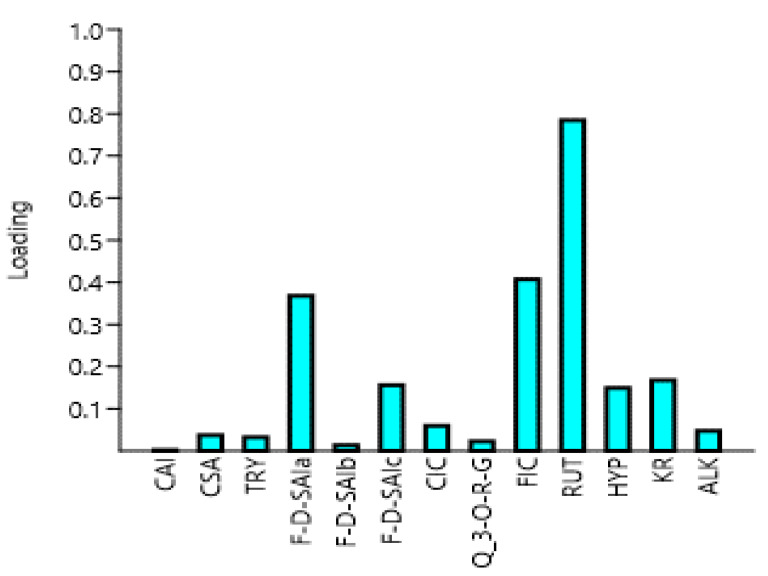
The loadings plot of the phenolic compounds and their percentage contribution to the total variations in the amaranth plant. CAI, caffeoylsaccharic acid isomer; CSA, coumaoryl saccharic acid; TRY, tryptophan; F-d-SAIa, feruloyl-d-saccharic acid isomer a; F-d-SAIb, feruloyl-d-saccharic acid isomer b; F-d-SAIc, feruloyl-d-saccharic acid isomer c; CIC, caffeoyl isocitrate; Q 3-*O*-RG, quercetin 3-*O*-rhamnosyl-rhamnosyl-glucoside; FIC, feruloyl isocitrate; RUT, rutin; HYP, hyperoside; KR, kaempferol rutinoside; ALK, alkaloid; TL, tender leaves; ML, mature leaves; LML, late maturity leaves; TG, tender grains; MG, mature grains; F, Flowers.

Principal component analysis (PCA) analysis in this study was used as a tool to optimize variables related to the quantities of polyphenols from different parts of the amaranth plant. The first principal component (PC1) in this study had the highest eigenvalue of 2.677, with a variance of 90.248, whereas the second principal component (PC2) had the second-highest eigenvalue, which was 2.238 with a variance of 8.4696 (Table 3 and Figure 4). Similarly, the third principal component (PC3) had an eigenvalue of 1.655 with a variance of 0.78548. The fourth principal component (PC4), however, had an eigenvalue of 0.898, which was less than one. Chatfield and Collin [40] and Hair et al. [41] stated that eigenvalue is only significant if it is more than one, and component loadings which are larger than ±0.30 are meaningful. Thus, the first three components (PC1, PC2, and PC3) are considered as important in the current study, as shown in Table 3. Rutin is believed to be the driving compound of the variability along the PC1 axis as it contains the highest component loading of 0.7381, as shown in Table 3 and Figure 3. On the other hand, the variability on the PC2 was influenced by caffeoyl isocitrate, quercetin 3-*O*-rhamnosyl-glucoside, feruloyl isocitrate, rutin, and alkaloid, which had a negative loading. While the variation on the PC3 was affected by caffeoyl isocitrate, rutin, kaempferol rutinoside, and alkaloid, which had a negative loading. To the best of our knowledge, no similar results were found in the literature.

**Figure 5:**
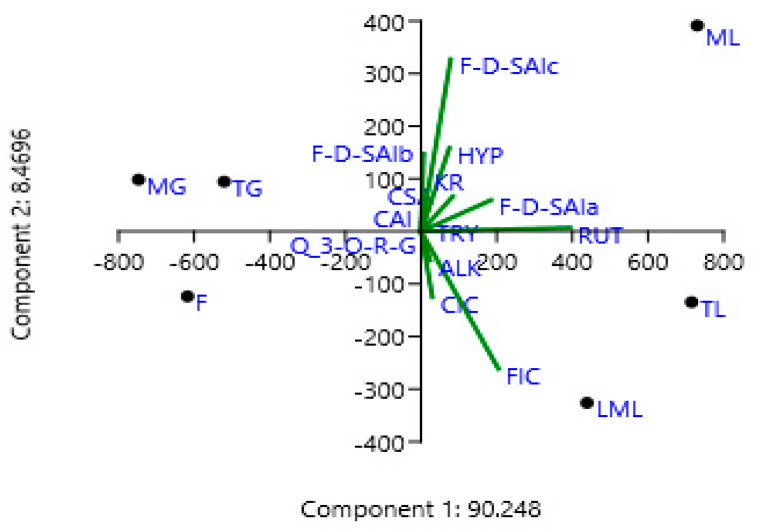
Principal component analysis (PCA) scatter plot of the phenolic compounds in amaranth. CAI, caffeoylsaccharic acid isomer; CSA, coumaoryl saccharic acid; TRY, tryptophan; F-d-SAIa, feruloyl-d-saccharic acid isomer a; F-d-SAIb, feruloyl-d-saccharic acid isomer b; F-d-SAIc, feruloyl-d-saccharic acid isomer c, CIC: Caffeoyl isocitrate, Q 3-*O*-RG: Quercetin 3-*O*-rhamnosyl-rhamnosyl-glucoside, FIC: Feruloyl isocitrate, RUT: Rutin, HYP: Hyperoside, KR: Kaempferol rutinoside, ALK: Alkaloid, TL: Tender leaves, ML: Mature leaves, LML: Late maturity leaves, TG: Tender grains, MG: Mature grains, F: Flowers.

As shown in Figure 5, the first principal component (PC1) on its own explained 90.248 of the variation, followed by PC2, 8.4696, and PC3, 0.78548%. Together they explained the variability of 99.50308%. Figure 2 indicates that a positive correlation exists between principal component 1 (PC1) and tryptophan, feruloyl-d-saccharic acid isomer a, feruloyl-d-saccharic acid isomer c, caffeoyl isocitrate, feruloyl isocitrate, rutin, hyperoside, kaempferol rutinoside, and alkaloid. Moreover, all these compounds were clearly shown to be related to tender, mature, and late maturity leaves and contributed high component loadings for component 1 (PC1). A negative correlation exists between principal component 2 (PC2) and caffeoylsaccharic acid isomer, coumaoryl saccharic acid, feruloyl-d-saccharic acid isomer b, quercetin 3-*O*-rhamnosyl-rhamnosyl-glucoside. Moreover, all these compounds clearly showed to be related to tender grains, mature grains, and flowers and contributed high component loadings for component 2 (PC2). Moreover, most of the phenolic compounds in the amaranth plant parts have been clustered on the principal component 1 (PC1) (Figure 5). However, no similar results were obtained from literature.

### 2.4. Hierarchical Clustering

**Figure 6:**
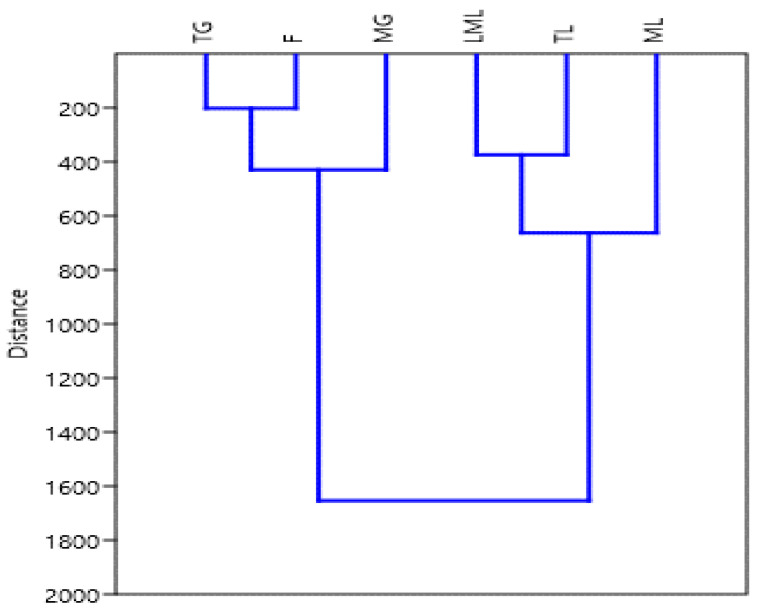
Hierarchical clustering of amaranth plant in different growth stage. TG, tender grains; F, flowers; MG, mature grains; LML, late maturity leaves; TL, tender leaves; ML, mature leaves.

Hierarchical cluster analysis (HCA) of different growth stages of amaranth plant parts has ranked them into four distinct groups (Figure 6). The tender and late maturity leaves were grouped on one side while the mature grain and flower were grouped in the other direction. Both mature leaves and tender grain stood on their own. These could be an indication that the tender and late maturity leaves contain similar kinds of polyphenols, while mature grains and flowers contain similar polyphenols.

### 2.5. Correlations

**Figure 7:**
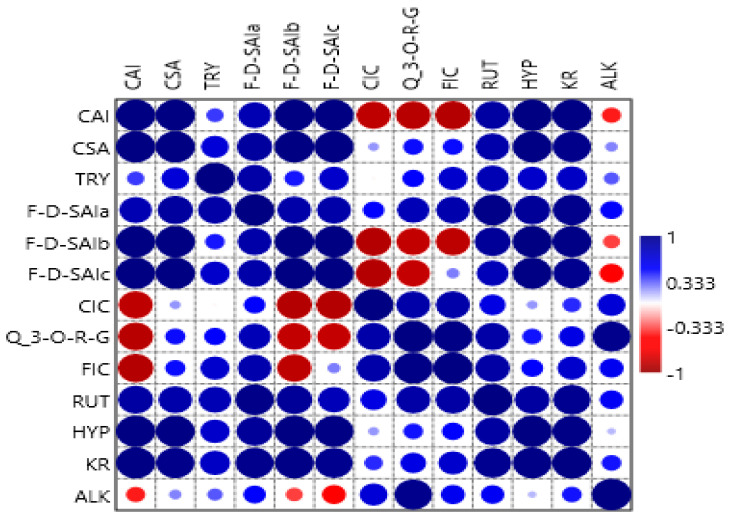
Pearson correlation plot for the phenolic compounds in amaranth plant. CAI, caffeoylsaccharic acid isomer; CSA: coumaoryl saccharic acid; TRY, tryptophan, F-d-SAIa, feruloyl-d-saccharic acid isomer a; F-d-SAIb, feruloyl-d-saccharic acid isomer b; F-d-SAIc, feruloyl-d-saccharic acid isomer c; CIC, caffeoyl isocitrate; Q 3-*O*-RG, quercetin 3-*O*-rhamnosyl-rhamnosyl-glucoside; FIC, feruloyl isocitrate; RUT, rutin; HYP, hyperoside, KR, kaempferol rutinoside; ALK, alkaloid, TL, tender leaves; ML, mature leaves; LML, late maturity leaves; TG, tender grains; MG, mature grains; F, Flowers.

The Pearson correlation plot for the phenolic compounds in the amaranth plant is presented in Figure 7. Caffeoylsaccharic acid isomer compound showed a strong positive relationship with coumaoryl saccharic acid, tryptophan, feruloyl-d-saccharic acid isomer a, feruloyl-d-saccharic acid isomer b, feruloyl-d-saccharic acid isomer c, rutin, hyperoside, and kaempferol rutinoside. Moreover, caffeoylsaccharic acid isomer compound showed a negative relationship with caffeoyl isocitrate, quercetin 3-*O*-rhamnosyl-rhamnosyl-glucoside, feruloyl isocitrate, and alkaloid. Coumaoryl saccharic acid compound showed a strong positive relationship with caffeoylsaccharic acid isomer, tryptophan, feruloyl-d-saccharic acid isomer a, feruloyl-d-saccharic acid isomer b, feruloyl-d-saccharic acid isomer c, caffeoyl isocitrate, quercetin 3-*O*-rhamnosyl-rhamnosyl-glucoside, feruloyl isocitrate, rutin, hyperoside, kaempferol rutinoside, and alkaloid. Tryptophan compound showed a strong positive relationship with caffeoylsaccharic acid isomer, coumaoryl saccharic acid, feruloyl-d-saccharic acid isomer a, feruloyl-d-saccharic acid isomer b, feruloyl-d-saccharic acid isomer c, caffeoyl isocitrate, quercetin 3-*O*-rhamnosyl-rhamnosyl-glucoside, feruloyl isocitrate, rutin, hyperoside, kaempferol rutinoside, and alkaloid. Feruloyl-d-saccharic acid isomer a compound showed a strong positive relationship with caffeoylsaccharic acid isomer, coumaoryl saccharic acid, tryptophan, feruloyl-d-saccharic acid isomer b, feruloyl-d-saccharic acid isomer c, caffeoyl isocitrate, quercetin 3-*O*-rhamnosyl-rhamnosyl-glucoside, feruloyl isocitrate, rutin, hyperoside, kaempferol rutinoside, and alkaloid. Feruloyl-d-saccharic acid isomer b and feruloyl-d-saccharic acid isomer c compounds showed a positive relationship with caffeoylsaccharic acid isomer, coumaoryl saccharic acid, tryptophan, feruloyl-d-saccharic acid isomer a, feruloyl-d-saccharic acid isomer c, and rutin. Moreover, feruloyl-d-saccharic acid isomer b, and feruloyl-d-saccharic acid isomer c compounds had a negative relationship with caffeoyl isocitrate, quercetin 3-*O*-rhamnosyl-rhamnosyl-glucoside, and alkaloid. Caffeoyl isocitrate and quercetin 3-*O*-rhamnosyl-rhamnosyl-glucoside compounds had a positive relationship with coumaoryl saccharic acid, tryptophan, feruloyl-d-saccharic acid isomer a, feruloyl isocitrate, rutin, hyperoside, kaempferol rutinoside, and alkaloid. Moreover, caffeoyl isocitrate and quercetin 3-*O*-rhamnosyl-rhamnosyl-glucoside showed a negative relationship with caffeoylsaccharic acid isomer, feruloyl-d-saccharic acid isomer b, and feruloyl-d-saccharic acid isomer c compounds. Feruloyl isocitrate compound showed a positive relationship with coumaoryl saccharic acid, tryptophan, feruloyl-d-saccharic acid isomer a, feruloyl-d-saccharic acid isomer c, feruloyl isocitrate, rutin, hyperoside, kaempferol rutinoside, and alkaloid. Moreover, feruloyl isocitrate compound showed a negative relationship with caffeoylsaccharic acid isomer, feruloyl-d-saccharic acid isomer b compounds. Rutin, hyperoside, kaempferol rutinoside compounds showed a positive relationship with caffeoylsaccharic acid isomer, coumaoryl saccharic acid, feruloyl-d-saccharic acid isomer a, feruloyl-d-saccharic acid isomer b, feruloyl-d-saccharic acid isomer c, feruloyl isocitrate, kaempferol rutinoside, and alkaloid compounds. No negative relationship was observed between rutin, hyperoside, kaempferol rutinoside, and other compounds. Alkaloid compound had a positive relationship with coumaoryl saccharic acid, tryptophan, feruloyl-d-saccharic acid isomer a, feruloyl isocitrate, rutin, hyperoside, and kaempferol rutinoside. Moreover, alkaloid had a negative relationship with caffeoylsaccharic acid isomer, feruloyl-d-saccharic acid isomer b, and feruloyl-d-saccharic acid isomer c compounds. Nsimba et al. [42] observed contrary results whereby a low correlation was observed between identified phenolic content in the amaranth plant. The correlation differences could be explained by individual methods and/or the presence of interfering substances.

## 3. Materials and Methods

### 3.1. Harvesting of Amaranth Leaves and Grains

*Amaranth cruentus* used in the present study were grown in a controlled field trial at North-West Province, South Africa. The area has mean temperatures of above 22 °C in summer and below 20 °C in winter and lies at latitude 25.6200° S and longitude 27.9800° E. In September 2019, under dry land conditions that receive a mean annual rainfall of less than 250 mm *Amaranth cruentus* used in the current study was planted. Amaranth leaves and grains were harvested at two stages; early and late harvest. Early harvest was done when the plant was 65 days and 120 days for the late harvest. Whereas flowers were harvested during late harvest at 120 days. Collected samples were thereafter separately dried in a well-ventilated laboratory and milled before being subjected to chemical analyses as described below.

### 3.2. Extraction of Phenolic Compounds

2 g of dry leaves, flowers, or grain material were accurately weighed out and extracted with 15 mL of 50% methanol/1% formic acid in water with vortexing, ultrasonication, and standing overnight. The amaranth extracts were then centrifuged, and the supernatant transferred to a glass vial before being subjected to LC-MS analysis.

### 3.3. LC-MS Analysis

The following chemicals were used: formic acid was purchased from Merck Pty Ltd. (Darmstadt, Germany), acetonitrile 200, and methanol 215 were purchased from Romil Ltd. (Waterbeach, Cambridge, UK), Catechin was from Sigma Aldrich (St. Louis, MO, USA).

A Waters Synapt G2 Quadrupole time-of-flight (QTOF) mass spectrometer (MS) connected to a Waters Acquity ultra-performance liquid chromatograph (UPLC) (Waters, Milford, MA, USA) with a photodiode array (PDA) detector was used for high-resolution UPLC-UV/MS analysis as described by Hassan et al., 2020. Electrospray ionization was used in negative mode with a cone voltage of 15 V, desolvation temperature of 275 °C, desolvation gas at 650 L/h, and the rest of the MS settings optimized for best resolution and sensitivity. Data were acquired by scanning from *m*/*z* 150 to 1500 *m*/*z* in resolution mode as well as in MSE mode. In MSE mode, two channels of MS data were acquired, one at a low collision energy (4 V) and the second using a collision energy ramp (20–60 V) to obtain fragmentation data as well. Leucine enkephalin was used as lock mass (reference mass) for accurate mass determination, and the instrument was calibrated with sodium formate. The separation was achieved on a Waters HSS T3, 2.1 × 100 mm, 1.7 μm column. An injection volume of 2 μL was used and the mobile phase consisted of 0.1% formic acid (solvent A) and acetonitrile containing 0.1% formic acid as solvent B. The gradient started at 100% solvent A for 1 min and changed to 28% B over 22 min in a linear way. It then went to 40% B over 50 s and a wash step of 1.5 min at 100% B, followed by re-equilibration to initial conditions for 4 min. The flow rate was 0.3 mL/min and the column temperature was maintained at 55 °C. Compounds were quantified in a relative manner against a calibration curve established by injecting a range of catechin standards from 0.5 to 100 mg/L catechin.

### 3.4. Statistical Analysis

Collected data were analysed using a one-way analysis of variance by statistical analysis software (SAS) version 9.2.1 (Raleigh, NC, USA) [43]. Duncan’s multiple range test was used for mean separation at a significance level of *p* < 0.05. Correlation among the phenolic compounds was done using the Principal Component Analysis (PCA) while the commercial statistical package PAST version 4.02 (Oslo, Norway) was used for multivariate cluster analysis and plotting.

## 4. Conclusions

It can be concluded that the type and quantity of the phenolic compounds in amaranth, varies across the plant parts and harvest stages. Tender and mature leaves have been shown to be richer in phenolic compounds than late maturity leaves, mature grains, tender grains, and flowers. Rutin compound was recorded as the most abundant phenolic compound in the amaranth plant, with the vegetative part having the biggest share. It can also be substantiated that the quantity of rutin compound fluctuated across growth stages, reaching its lowest in the tender grain stage. The amaranth plant investigated in this study showed the presence of diverse phenolic compounds. These phenolic compounds have health benefits in human beings, which may aid in fighting against different diseases. Therefore, results obtained from this study indicate the importance of amaranth as a promising source of phenolic compounds that will help in developing new opportunities for their use in the food and pharmaceutical industries. However, future studies are recommended for individual compound purification and their mechanisms of action, which will provide a better understanding of antioxidant activity nature before they can be included in human diets and used in the industrial manufacture of drugs.

## Figures and Tables

**Table 1:** Phenolic compounds identified in amaranth plant parts using LC-MS analysis.

Compound No	Formula [MH]	RT (min)	M − H^−^ (*m*/*z*)	Mass Error (ppm)	Product Ions (*m*/*z*)	UV (λ, nm)	Tentative Identification
**1**	C_15_H_15_O_11_	6.915	371.063	0.8	209.191.151.85	324	Caffeoylsaccharic acid isomer
**2**	C_15_H_15_O_11_	7.35	371.061	−1.9	209.85	Nd	unknown
**3**	C_15_H_15_O_11_	8.01	371.06	Nd	Nd	Nd	unknown
**4**	C_15_H_15_O_11_	8.54	371.061	Nd	Nd	Nd	unknown
**5**	C_15_H_15_O_10_	8.98	355.069	−1.4	209.191.147.85	312	Coumaoryl saccharic acid
**6**	C_11_H_11_N_2_O_2_	9.14	203.826	Nd	Nd	Nd	Tryptophan
**7**	C_16_H_17_O_11_	9.62	385.076	−4.2	209.191.173	324	unknown
**8**	C_16_H_17_O_11_	10.42	385.077	−1.6	209.191.147.85	327	Feruloyl-d-saccharic acid isomer a
**9**	C_16_H_17_O_11_	10.89	385.078	−4.7	209.191.147.129.85	327	Feruloyl-d-saccharic acid isomer b
**10**	C_16_H_17_O_11_	11.35	385.076	−5.2	209.191.147.129.85	327	Feruloyl-d-saccharic acid isomer c
**12**	C_15_H_13_O_10_	13.08	353.053	2.0	191.173.155.111	327	Caffeoyl isocitrate
**13**	C_22_H_29_O_14_	13.95	517.157	2.9	459.193.179.176.160	324	unknown
**14**	C_5_H_17_N_6_O_9_	14.53	305.107	1.1	305.97	271.328	unknown
**15**	C_19_H_23_O_12_	15.03	443.119	0.7	267.193	324	unknown
**16**	C_33_H_39_O_20_	15.49	755.205	−1.6	611.300.271	332	Quercetin 3-*O*-rhamnosyl-rhamnosyl-glucoside
**17**	C_16_H_15_O_10_	16.35	367.068	1.1	173.154.111	328	Feruloyl isocitrate
**18**	C_27_H_29_O_16_	17.09	609.143	−1.8	300.271	339	Rutin
**19**	C_21_H_19_O_12_	17.46	463.086	−3.0	300.271	348	Quercetin galactoside (Hyperoside)
**20**	C_27_H_29_O_15_	18.65	593.151	0.5	285.255	339	Kaempferol rutinoside
**21**	C_34_H_45_O_14_	22.18	677.283	2.4	645.617.489.279.152	312	unknown
**22**	C_18_H_18_NO_4_	22.54	312.124	1	190.178.148.135	289.318	Alkaloid

Abbreviations: Nd = not detected.

**Table 2:** Quantified individual phenolic compounds in amaranth plant parts (mg/kg).

Compound.	TL	ML	LML	TG	MG	F	SEM	*p*
Caffeoylsaccharic acid isomer	19.0 ^b^	45.8 ^a^	7.9 ^c^	0.00 ^d^	0.00 ^d^	0.00 ^d^	0.005	0.0001
Coumaoryl saccharic acid	57.0 ^b^	106.2 ^a^	33.3 ^c^	0.00 ^f^	2.1 ^e^	14.7 ^d^	0.005	0.0001
Tryptophan	87.8 ^a^	67.3 ^b^	37.6 ^d^	27.3 ^e^	38.9 ^c^	15.0 ^f^	0.006	0.0001
Feruloyl-d-saccharic acid isomer a	514.9 ^b^	547.8 ^a^	328.6 ^c^	1.2 ^f^	5.3 ^e^	13.7 ^d^	0.000	0.0001
Feruloyl-d-saccharic acid isomer b	300.2 ^b^	602.1 ^a^	127.3 ^c^	0.00 ^e^	0.00 ^e^	2.2 ^d^	0.139	0.0001
Feruloyl-d-saccharic acid isomer c	300.2 ^b^	602.1 ^a^	127.3 ^c^	0.00 ^e^	0.00 ^e^	2.21 ^d^	0.139	0.0001
Caffeoyl isocitrate	177.6 ^b^	135.9 ^c^	317.0 ^a^	0.00 ^e^	6.4 ^d^	0.00 ^e^	0.005	0.0001
Quercetin 3-*O*-rhamnosyl-rhamnosyl-glucoside	79.3 ^a^	50.8 ^c^	73.0 ^b^	0.00 ^e^	0.90 ^d^	0.00 ^e^	0.005	0.0001
Feruloyl isocitrate	683.1 ^a^	335.4 ^c^	621.7 ^b^	2.8 ^e^	13.1 ^d^	2.0 ^f^	0.000	0.0001
Rutin	1223 ^b^	1290 ^a^	1042 ^c^	26 ^f^	342 ^d^	252 ^e^	0.000	0.0001
Quercetin galactoside	206.5 ^b^	390.8 ^a^	130.5 ^c^	0.00 ^f^	18.5 ^d^	5.41 ^e^	0.005	0.0001
Kaempferol rutinoside	193.8 ^b^	311.9 ^a^	164.0 ^c^	1.8 ^e^	19.9 ^d^	19.0 ^d^	3.400	0.0001
Alkaloid	147.33 ^a^	87.58 ^b^	101.12 ^c^	2.88 ^f^	15.60 ^e^	142.01 ^d^	0.005	0.0001

Mean values of individual polyphenols a, b, c, d, e, f: Means in the same row with different superscripts are significantly different at (*p* < 0.05). TL, tender leaves; ML, mature leaves; LML, late maturity leaves; TG, tender grains; MG, mature grains; F, flowers.

**Table 3:** Principal component analysis of the phenolic compounds in the amaranth plant, showing their percentage contribution to the total variations.

	PC1	PC2	PC3
Caffeoylsaccharic acid isomer	0.0198	0.0455	0.01407
Coumaoryl saccharic acid	0.0480	0.0822	0.01525
Tryptophan	0.0298	0.0097	0.09928
Feruloyl-d-saccharic acid isomer a	0.3464	0.1021	0.48839
Feruloyl-d-saccharic acid isomer b	0.1528	0.2751	0.14245
Feruloyl-d-saccharic acid isomer c	0.2771	0.5404	0.27678
Caffeoyl isocitrate	0.1369	−0.2831	−0.19955
Quercetin 3-*O*-rhamnosyl-glucoside	0.0473	−0.0605	0.05164
Feruloyl isocitrate	0.3760	−0.6489	0.50101
Rutin	0.7381	−0.0267	−0.5927
Quercetin galactoside (Hyperoside)	0.1861	0.2895	0.05488
Kaempferol rutinoside	0.1589	0.14688	−0.02701
Alkaloid	0.047297	−0.11011	−0.22508
Eigenvalue	2.6770	2.238	1.6550
% variance	90.248	8.4696	0.78548
Cumulative %	90.248	98.7176	99.50308
